# Inverted bulk-heterojunction solar cell with cross-linked hole-blocking layer

**DOI:** 10.1016/j.orgel.2014.02.009

**Published:** 2014-05

**Authors:** Yasemin Udum, Patrick Denk, Getachew Adam, Dogukan H. Apaydin, Andreas Nevosad, Christian Teichert, Matthew. S. White, Niyazi. S. Sariciftci, Markus C. Scharber

**Affiliations:** a Institute for Organic Solar Cells, Johannes Kepler University Linz, Altenbergerstrasse 69, 4040 Linz, Austria; b Institute of Science and Technology, Department of Advanced Technologies, Gazi University, 06570 Ankara, Turkey; c Institute of Physics, Montanuniversitaet Leoben, Franz-Josef-Straße 18, 8700 Leoben, Austria

**Keywords:** Organic solar cell, Device design, Electrical contact, Stability

## Abstract

We have developed a hole-blocking layer for bulk-heterojunction solar cells based on cross-linked polyethylenimine (PEI). We tested five different ether-based cross-linkers and found that all of them give comparable solar cell efficiencies. The initial idea that a cross-linked layer is more solvent resistant compared to a pristine PEI layer could not be confirmed. With and without cross-linking, the PEI layer sticks very well to the surface of the indium–tin–oxide electrode and cannot be removed by solvents used to process PEI or common organic semiconductors. The cross-linked PEI hole-blocking layer functions for multiple donor–acceptor blends. We found that using cross-linkers improves the reproducibility of the device fabrication process.

## Highlights

• Inverted bulk heterojunction solar cell.
• Work function modification of ITO.
• Improved stability under ambient conditions compared to conventional devices.

## Graphical abstract

**Figure:**
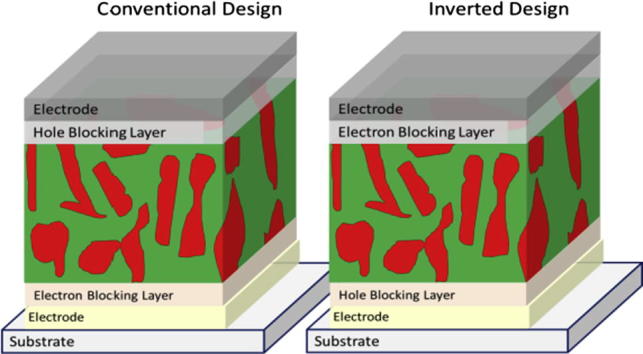

## 1 Introduction

**Fig. 1:**
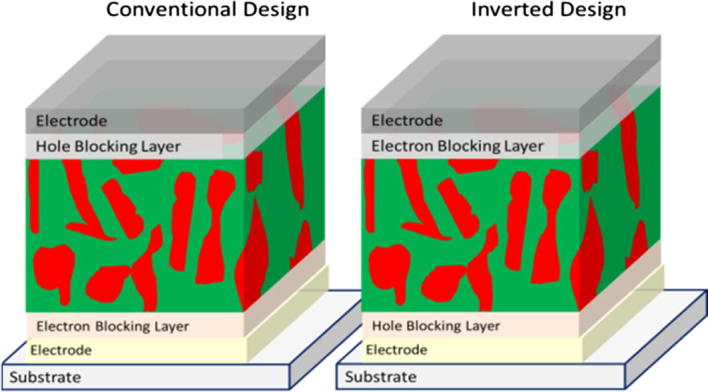
Schematic device structure of a conventional and an inverted bulk-heterojunction solar cell.

Within the field of organic solar cells, the conventional bulk-heterojunction device (see Fig. 1) comprises a substrate covered with a transparent conductive oxide, an electron blocking layer, a photoactive layer, in some case a hole blocking layer and a metal electrode [1]. Often indium-tin-oxide is used as the transparent electrode, and poly(3,4-ethylenedioxythiophene) poly(styrenesulfonate) (PEDOT:PSS) is applied as electron blocking layer. TiO*_x_*, CsCO_3_ or LiF are examples of hole blocking layers used in devices based on conventional design [1–4].

The photoactive bulk-heterojunction layer is located between the hole and electron blocking layers with domains of acceptor material dispersed in a donor matrix. Devices are completed with a non-transparent electrode made of a low work function metal like calcium or aluminum. Under ambient conditions these low work function metals typically form non-conductive oxides leading to a rapid degradation of the solar cell performance [5,6]. Therefore rigorous encapsulation of these devices is required using costly packaging materials with very low oxygen and water permeation.

Inverted bulk-heterojunction solar cells (see Fig. 1) are based on an opposite layer sequence having the hole blocking layer between the transparent electrode and the photoactive layer. The top contact uses an electron blocking layer and a high work-function, air-stable material like silver or gold. The first inverted organic solar cell was prepared by Shirakawa et al. [7] applying a compact ZnO hole-blocking layer and a gold layer as top electrode. Various other oxides (such as solution processed TiO*_x_* [8] and ZnO[9]), organic or polymeric interlayers[10] or CsCO_3_[11] were reported as efficient hole-blocking layers on ITO. As electron blocking layers, PEDOT:PSS and different p-type oxides like MoO_3_ or V_2_O_5_ have been applied [12]. Substantially improved stabilities under ambient conditions were reported for inverted devices while the power conversion efficiencies of conventional and inverted bulk-heterojunction solar cells made of the same absorber blend were found to be comparable. In addition, Krebs et al. demonstrated that inverted cells can be manufactured solely via solution processing making the inverted design especially interesting for large area coating [13].

The amine-rich polymer polyethylenimine (PEI) may also serve as a hole-blocking layer in organic solar cells, as published in the patent application (US2008/0264499 A1, now patent US8242356) in 2008. Recently Zhou et al. [14] reported the use of PEI as thin interlayer between the photoactive layer and an electrode material. The authors demonstrated that applying PEI on various different materials shifts the work function and leads to an electron selective contact. This system allows air-stable materials to be used in place of reactive Ca or LiF. Here we report a similar approach applying a cross-linked PEI layer on top of ITO. The motivation for this work was to show that cross-linked PEI-layers were insoluble and more robust, which may be used in solution processed multi-junction devices. Cross-linking PEI is very well known in the framework of membrane science and we have selected 5 different ether-based cross-linkers [15]. After some optimization, high performance devices were made with all of the selected cross-linkers. We found that all PEI films, with and without cross-linking, were robust to various solvent treatments. However, the developed process for cross-linked PEI interlayers is easy and insensitive to small variations in the processing conditions, which is essential for processing very thin (<10 nm) layers.

## 2 Experimental

Regio-regular poly(3-hexylthiophen-2,5-diyl) (P3HT) was purchased from Rieke Metals, Poly[[4,8-bis[(2-ethylhexyl)oxy]benzo[1,2-b:4,5-b′]dithiophene-2,6-diyl][3-fluoro-2-[(2-ethylhexyl)carbonyl]thieno[3,4-b]thiophenediyl]] (PTB7) was purchased from 1-Material, PC60BM was purchased from SolenneBV, polyethylenimine branched (Mw = 800 g/mol) and the cross-linkers glycerol diglycidyl ether (CL1), bisphenol A diglycidyl ether (CL2), 1,4-butanediol diglycidyl ether (CL3), poly(propylene glycol) diglycidyl ether (CL4) and trimethylolethane triglycidyl ether (CL5) were purchased from Sigma Aldrich [16–19]. Materials were used as received without further purification. P3HT and PC60BM were dissolved together in chlorobenzene (1:0.7 wt%). PEI was dissolved in butanol (0.27 mg/ml). PEI and crosslinkers were also dissolved in butanol at following weight ratios per milliliter of butanol:

PEI/Glycerol diglycidyl ether (0.27 mg/0.44 mg), PEI/Bisphenol A diglycidyl ether (0.27 mg/0.74 mg), PEI/1,4-Butanediol diglycidyl ether (0.27 mg/0.44 mg), PEI/Poly(propylene glycol) diglycidyl ether (0.27 mg/0.82 mg) and PEI:Trimethylolethane Triglycidyl Ether (0.27 mg/0.42 mg). Devices were fabricated on indium tin oxide (ITO) coated glass substrates with a sheet resistance 15 Ω/square. The substrates were cleaned by sonication in acetone and isopropanol for 15 min. PEI-solution and the PEI:Crosslinker solutions were spin coated on the ITO substrates at 2000 rpm and then annealed at 105 °C on a hotplate for 10 min. Then the blend of P3HT and PCBM was spin coated at 1500 rpm. and annealed at 110 °C for 5 min. The active layer thickness was ∼80 nm. Finally, an 8 nm MoO_3_ layer and a 100 nm Ag layer were evaporated through a shadow mask to define the active area (∼9 mm^2^) and form the hole-collecting electrode. Conventional bulk heterojunction solar cells were prepared by spin-coating a 50 nm thick PEDOT:PSS layer (Clevios P 4083) on top of a cleaned glass substrates coated with ITO. The active layer was deposited and annealed as described above. The device was finished by evaporating 0.8 nm of LiF and 80 nm of Aluminium through a shadow mask. Devices were characterized on a Steuernagel solar simulator and external quantum efficiencies were measured on a home-built setup based on a white-light lamp, a monochromator and a lock-in detection. Device lifetime studies were performed by storage of the devices in ambient air conditions, with periodic measurements under white light.

Kelvin probe force microscopy (KPFM) was employed to confirm the intended changes in work function of the PEI interlayers. KPFM is a variant of atomic force microscopy (AFM) that allows mapping the local contact potential difference between an AFM tip and the sample surface [20,21]. The technique therefore yields the local work function and is also applicable to organic thin films [22,23]. The KPFM investigations were performed on an MFP-3D atomic force microscope from AsylumResearch. As probes, TiN coated NSG 03 silicon cantilevers from NT-MDT were employed. These probes feature a tip radius below 35 nm, a typical resonance frequency of 90 kHz and a spring constant of 1.74 N/m. For the TiN coated tip, a work-function of approximately 5 eV was estimated [24]. A profilometer (DektakXT, Bruker) was used to determine the layer thickness of the cross-linked PEI layers. After scraching the layer with a sharp needle the profile was scanned with the dektak tip.

## 3 Results and discussion

**Fig. 2:**
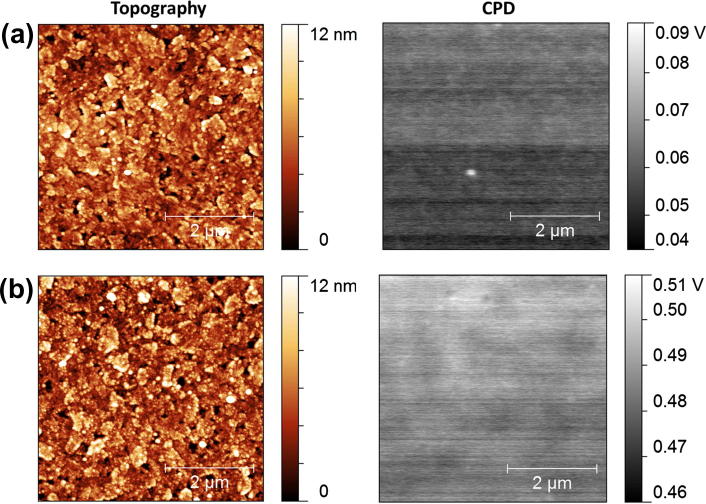
5 by 5 μm topography images and corresponding contact potential difference maps of ITO on glass (a) and Glass/ITO/PEI + glycerol diglycidyl ether (CL1) (b).

The work function of the different bottom electrodes was studied using Kelvin probe force microscopy. Typical Kelvin probe pictures of ITO and an ITO layer covered with cross-linked PEI are shown in Fig. 2. The complete set of topographies and CPD scans can be found in the Supplementary content. Average values for the contact voltage potentials of all investigated devices are summarized in Table 1. The measurements show that the applied PEI- and cross-linked PEI-layers reduce the work function of the ITO by about 400–500 mV. The consistently high V_OC_ and FF in our solar cells show that this large shift is sufficient to convert the ITO into a high performance hole-blocking electrode.

The topographies of all investigated surfaces were found to be very similar. Please note, that the CPD fluctuations in the KPFM images, presented in Fig. 2, as for the other samples investigated are in the range of 0.05 V. These fluctuations are mainly caused by slight changes of the AFM-tip properties during scanning. This confirms the high homogeneity in work function of the prepared films.

The layer thickness measurements performed with a profilometer confirm that the PEI layers are very thin. Thickness in the range of 3–5 nm were observed. Due to the roughness of the investigated layers, a precise determination of the layer thickness was not possible.

**Fig. 3:**
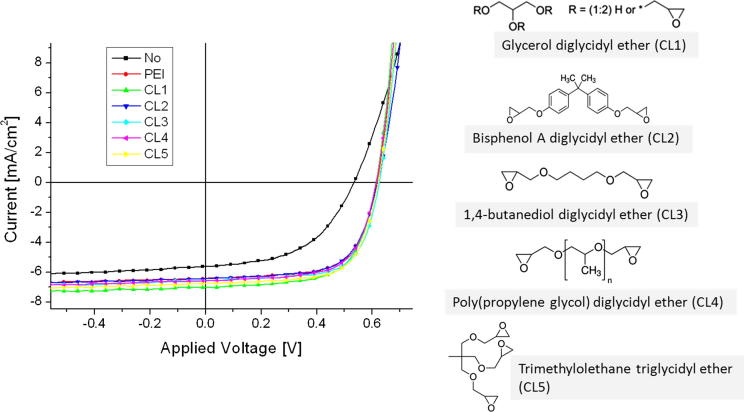
Current–voltage curves of different P3HT-PCBM solar cells with different interlayers between ITO and photoactive layer and the chemical structures of the investigated cross-linkers.

The current–voltage curves of typical solar cells, with and without PEI-interlayers, are plotted in Fig. 3, and the important device parameters are summarized in Table 2. All short circuit currents were corrected for the spectral mismatch of the used solar simulator by determining the external quantum efficiency of the solar cells. The recorded spectra are shown in the Supplementary content.

All devices with a thin PEI interlayer between ITO and the active layer show a comparable performance. Applying the same processing conditions, we found no significant difference between the five tested cross-linkers in our experiments. It is interesting to note that even the device with a MoO_3_ electron blocking layer and no hole-blocking layer exhibits a diode-like current–voltage curve and a power conversion efficiency of ∼1.6%. This suggests that only one optimized contact is sufficient for a working bulk-heterojunctions solar cell.

We then exposed the various PEI layers (on ITO) to several different solvent treatments before deposition of the active layer. This was primarily to test the robustness of the PEI to potential processing solvents for high throughput manufacturing and even tandem solar cells. We find that soaking the processed PEI layers in butanol (the same solvent used to deposit the PEI) for 30 min has essentially no impact on the device performance. Exposing the PEI-layers to solvents for the organic semiconductors like chlorobenzene for 2 h, also showed minimal effects in the final performance. The device parameters are statistically indistinguishable with or without the solvent treatments, and fill-factors between 60% and 67% and an open circuit voltages ∼620 mV were obtained. Device data can be found in the Supplementary content, Table S1. It is even more surprising that pure PEI and the cross-linked PEI layers were equally robust to these solvent treatments. It appears that there is a strong interaction between ITO and PEI leading to an immobilization of the PEI-polymer on the ITO surface.

The cross-linked PEI-layers also work for other polymer-fullerene blends different to P3HT/PCBM. We demonstrate their use with Poly[[4,8-bis[(2-ethylhexyl)oxy]benzo[1,2-b:4,5-b′]dithiophene-2,6-diyl][3-fluoro-2-[(2-ethylhexyl)carbonyl]thieno[3,4-b]thiophenediyl]] (PTB7)-PCBM devices prepared following the procedure described in [10], resulting in devices with PCE > 6% (see Supplementary content, Fig. S3). In general the power conversion efficiencies of conventional and inverted devices were found to be very similar. In some cases slightly different processing conditions of the active layer were required for the preparation of high performance inverted solar cells. This may be related to a vertical phase separation of the donor and acceptor. For the conventional device, an acceptor rich phase near the low work-function electrode is preferable and the top surface of the photoactive layer of an inverted solar cell should be predominantly made of donor material. This can be achieved by using different solvents or solvent mixtures or by working with processing additives [25].

**Fig. 4:**
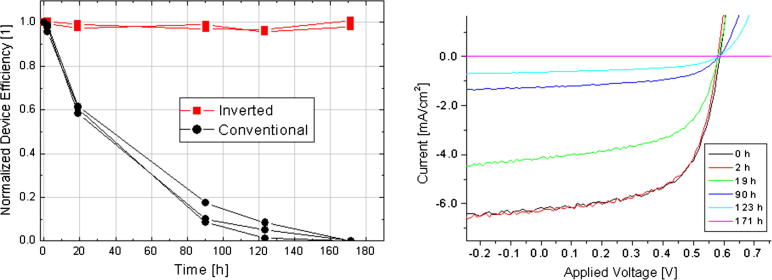
Power conversion efficiencies of conventional and inverted P3HT-PCBM devices stored under ambient conditions (left). Right: Current–voltage curves measured on a conventional device during the degradation process.

The inverted devices exhibit significantly better stability under ambient conditions. In Fig. 4, the power conversion efficiencies measured after different storage times of three conventional and two inverted devices are plotted. While storage in air leads to a rapid degradation due to oxidation of the aluminum electrode in conventional devices, the performance of the inverted devices remains essentially unchanged. Because these inverted cells are more stable in air, the costly encapsulation requirements may be relaxed for solar cell manufacturing.

In summary, we have developed a cross-linked hole-blocking layer for inverted bulk heterojunction solar cells. We found that different cross-linkers can be added to PEI resulting in a very similar overall device performance. The new hole-blocking layer works for various different polymer-fullerene blends. Its processing is simple and allows device manufacturing with very high yield. This may be highly relevant for the development of next-generation organic tandem solar cells. The envisioned improvement of the mechanical and chemical properties of cross-linked PEI-layers on ITO was not observed in our experiments. Our results suggest that PEI adheres very well to ITO and cannot be removed by common organic solvents.

## Figures and Tables

**Table 1:** Average contact potential differences of different layers.

Layer stack	Contact potential difference (V)
Glass/ITO	0.05
Glass/ITO/PEI	0.39
Glass/ITO/PEI + glycerol diglycidyl ether (CL1)	0.49
Glass/ITO/PEI + bisphenol A diglycidyl ether (CL2)	0.57
Glass/ITO/PEI + 1,4-butanediol diglycidyl ether (CL3)	0.55
Glass/ITO/PEI + poly(propylene glycol) diglycidyl ether (CL4)	0.53
Glass/ITO/PEI + trimethylolethane triglycidyl ether (CL5)	0.47

**Table 2:** Solar cell parameters measured under illumination.

Sample	*J*_sc_ (mA/cm^2^)	*V*_oc_ (mV)	FF (%)	Efficiency (%)
Conventional DeviceITO/PEDOT:PSS/P3HT:PC_61_BM/LiF/Alu	6.3	590	61	2.3
ITO/P3HT:PC_61_BM/MoO_3_/Ag	5.7	550	52	1.6
ITO/PEI/P3HT: PC_61_BM /MoO_3_/Ag	6.4	620	67	2.7
ITO/PEI:CL1/P3HT:PC_61_BM/MoO_3_/Ag	6.9	620	67	2.9
ITO/PEI:CL2/P3HT:PC_61_BM/MoO_3_/Ag	6.6	620	64	2.6
ITO/PEI:CL3/P3HT:PC_61_BM/MoO_3_/Ag	6.6	630	69	2.9
ITO/PEI:CL4/P3HT:PC_61_BM/MoO_3_/Ag	6.4	620	68	2.7
ITO/PEI:CL5/P3HT:PC_61_BM/MoO_3_/Ag	6.8	620	68	2.9

## Appendix A Supplementary material

Supplementary data 1 This supplementry contain Figs. S1–S3, Table S1.
