# Supplementary material for: Inverted bulk-heterojunction solar cell with cross-linked hole-blocking layer
Source: Org Electron. 2014 May;15(5):997–1001. doi: 10.1016/j.orgel.2014.02.009 (PMC4010259; doi:10.1016/j.orgel.2014.02.009)
Supplement: Supplementary data 1 — This supplementry contain Figs. S1–S3, Table S1. [file mmc1.docx]

[Supplementary content](http://www.sciencedirect.com/science/article/pii/S1566119913005582#MMCvFirst)

Inverted bulk-heterojunction solar cell with cross-linked hole-blocking layer

Yasemin Udum^1,2^, Patrick Denk^1^, Getachew Adam^1^, Dogukan H. Apaydin^1^, Andreas Nevosad^3^, Christian Teichert^3^, Matthew. S. White^1^, Niyazi. S. Sariciftci^1^, Markus C. Scharber^*1^

^1^Institute for Organic Solar Cells, Johannes Kepler University Linz, Altenbergerstrasse 69, 4040 Linz, Austria

^2^Institute of Science and Technology, Department of Advanced Technologies, Gazi University, 06570 Ankara, Turkey

^3^Institute of Physics, Montanuniversitaet Leoben, Franz-Josef-Straße 18, A-8700 Leoben, Austria

In Figure S1 the external quantum efficiency (EQE) spectra of different solar cells are shown. Integration of the EQE spectra multiplied by the solar spectrum yields the short circuit current under standard illumination conditions. Calculated short circuit currents are shown the inset of Figure S1.

Figure S1: External Quantum Efficiency Spectra of bulk heterojunction solar cells with different interlayers between ITO and the photoactive layer (P3HT/PC_60_BM)

In Figure S2 topographies and contact potential difference (CPD) scans are shown.


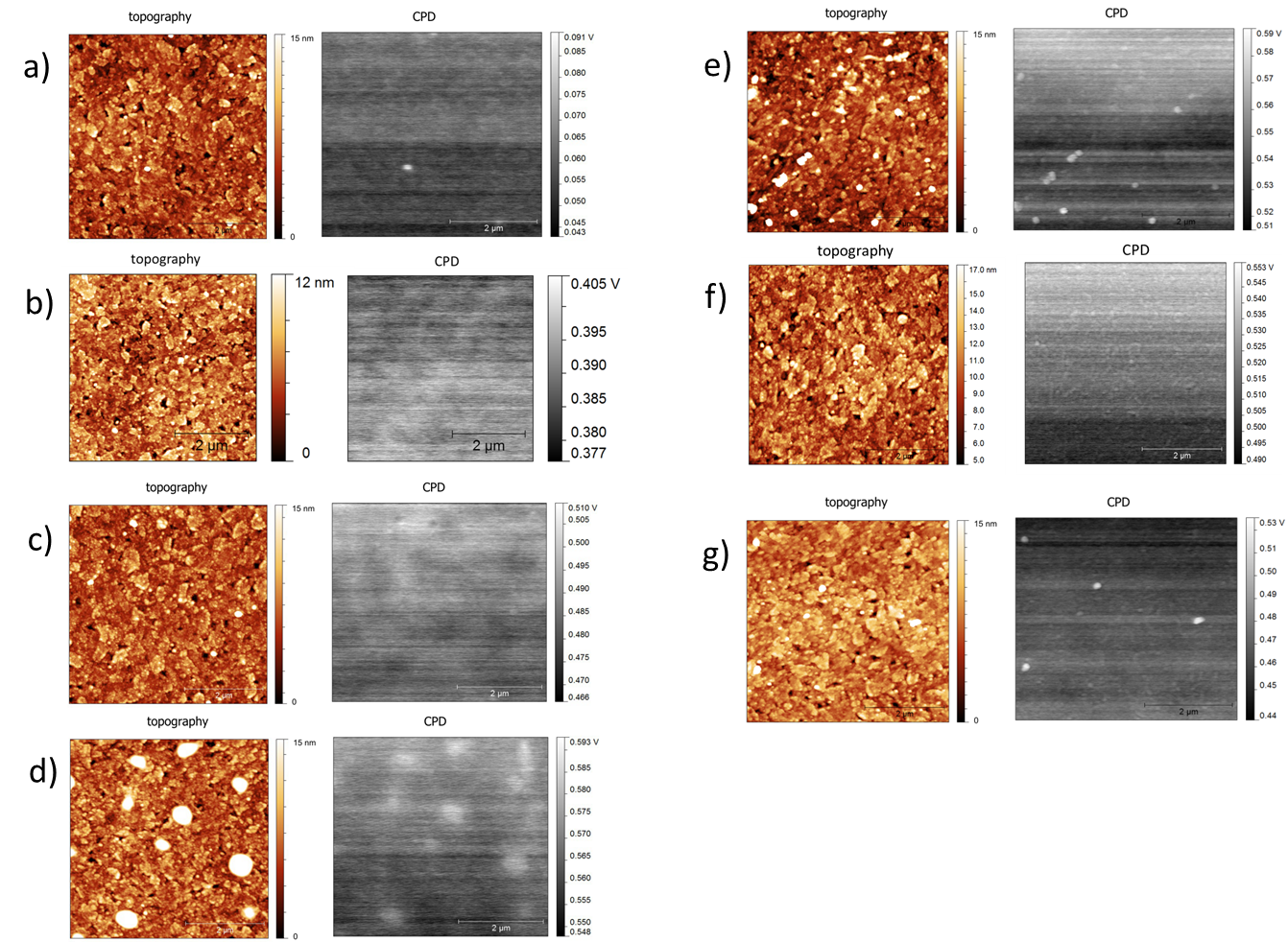


Figure S2: Topography and contact potential difference (CPD) of a) Glass/ITO, b) Glass/ITO/PEI, c) Glass/ITO/PEI + glycerol diglycidyl ether (CL1), d) Glass/ITO/PEI + bisphenol A diglycidyl ether (CL2), e) Glass/ITO/PEI + 1,4-butanediol diglycidyl ether (CL3), f) Glass/ITO/PEI + poly(propylene glycol) diglycidyl ether (CL4), g) Glass/ITO/PEI + trimethylolethane triglycidyl ether (CL5)

The bright spots observed on some surfaces are most likely related to particles which were picked up from the surface by the microscope tip. This reduced the lateral resolution and changed the tip work function.

Examples of solvent treatments of PEI and cross-linked PEI layers are summarized in Table S1. A longer exposure of the interlayer to butanol or chlorobenzene does not degrade the functionality of the layer. The short circuit current values given in Table S1 are not corrected for the spectral mismatch and active area variations occurring during device processing.

| **Sample** | **Jsc**  **[mA/cm^2^]** | **Voc**  **[V]** | **FF** | **Eff.**  **[%]** |
| --- | --- | --- | --- | --- |
| PEI+CL1 - n-Butanol spin-coated on top of the interlayer | 6.7 | 0.62 | 0.63 | 3.26 |
| PEI+CL1 - Chlorobenzene spin-coated on top of the interlayer | 6.1 | 0.61 | 0.63 | 2.9 |
| PEI+CL1 – layer dipped in n-Butanol for 5 seconds | 6.4 | 0.61 | 0.64 | 3.1 |
| PEI+CL1 – layer dipped in n-Butanol for 15 minutes | 7.6 | 0.62 | 0.65 | 3.81 |
| PEI+CL1 – layer dipped in n-Butanol for 1 hour | 7.2 | 0.61 | 0.65 | 3.56 |
| PEI sonication in n-Butanole for 30 min | 7.2 | 0.62 | 0.68 | 3.79 |
| PEI sonication in n-Butanole for 1 hour | 7.2 | 0.63 | 0.67 | 3.76 |
| PEI dipping in water for 2 hour | 7.5 | 0.61 | 0.57 | 3.27 |
| PEI dipping in chlorobenzene for 2 hour | 6.8 | 0.62 | 0.68 | 3.62 |

Table S1

In Figure S3 a current-voltage curve and the external quantum efficiency spectrum of a PTB7/PC_71_BM device are shown.


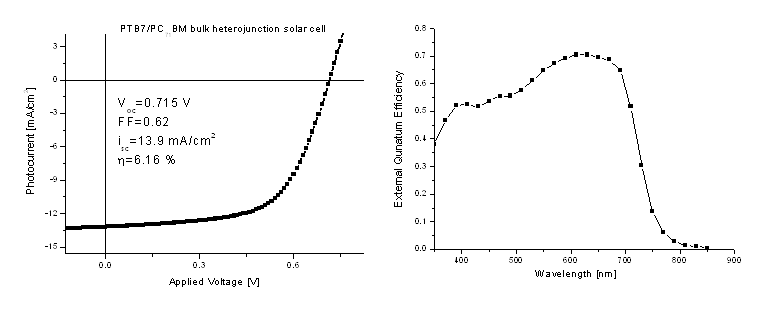


Figure S3: Current-voltage curve (left) and EQE spectrum (right) of a PTB7/PC_71_BM device.
